# Effects of living near a new urban motorway on the travel behaviour of local residents in deprived areas: Evidence from a natural experimental study

**DOI:** 10.1016/j.healthplace.2016.11.008

**Published:** 2017-01

**Authors:** Louise Foley, Richard Prins, Fiona Crawford, Shannon Sahlqvist, David Ogilvie, on behalf of the M74 study team

**Affiliations:** a MRC Epidemiology Unit & UKCRC Centre for Diet and Activity Research (CEDAR), School of Clinical Medicine, University of Cambridge, Box 285, Cambridge Biomedical Campus, Cambridge CB2 0QQ, United Kingdom; b NHS Greater Glasgow & Clyde and Glasgow Centre for Population Health, Third floor, Olympia Building, Bridgeton Cross, Glasgow G40 2QH, United Kingdom; c Centre for Physical Activity and Nutrition Research (C-PAN), School of Exercise and Nutrition Sciences, Deakin University, 75 Pigdons Road, Waurn Ponds, Victoria 3216, Australia

**Keywords:** Road, Automobiles, Active travel, Transportation, Natural experimental study

## Abstract

We evaluated the effects of a new motorway built through deprived neighbourhoods on travel behaviour in residents. This natural experiment comprised a longitudinal cohort (n=365) and two cross-sectional samples (baseline n=980; follow-up n=978) recruited in 2005 and 2013. Adults from one of three study areas - surrounding the new motorway (South), an existing motorway (East), or no motorway (North) - completed a previous day travel record. Adjusted two-part regression models examined associations between exposure and outcome. Compared to the North, cohort participants in the South were more likely to undertake travel by any mode (OR 2.1, 95% CI 1.0–4.2) at follow-up. Within the South study area, cohort participants living closer to a motorway junction were more likely to travel by any mode at follow-up (OR 4.7, 95% CI 1.1–19.7), and cross-sectional participants living closer were more likely to use a car at follow-up (OR 3.4, 95% CI 1.1–10.7), compared to those living further away. Overall, the new motorway appeared to promote travel and car use in those living nearby, but did not influence active travel. This may propagate socioeconomic inequalities in non-car owners.

## 1 Introduction

Social, physical and broader economic environments are recognised as key influences on people's behaviour and health (Sallis et al., 2006). While cross-sectional studies indicate associations between features of the built environment and both physical activity (Saelens and Handy, 2008) and sedentary behaviour (Koohsari et al., 2015), there is little longitudinal evidence to show whether and how changing the environment changes these behaviours (National Institute for Health and Clinical Excellence (NICE), 2008; National Institute for Health and Clinical Excellence (NICE), 2014.

Recently, research and policy attention has been drawn to the potential of active travel (walking or cycling for transport) to contribute to daily physical activity (Sahlqvist et al., 2013; Foley et al., 2015) and promote good health (Kelly et al., 2014). Active travel can be made a habitual, sustainable part of everyday life, as well having important co-benefits such as helping to curb carbon emissions through reduced reliance on motorised transport (Sallis et al., 2015). In tandem, reducing car use has been identified as an important policy objective (British Medical Association, 2012) because of the relationship between motor vehicle use and poor health (Sugiyama et al., 2016) via physical inactivity, air pollution and injuries from road traffic accidents (Woodcock et al., 2007). Reducing car use has also been promoted on equity grounds. People from lower socioeconomic backgrounds are less likely to have access to motor vehicles, yet deprived areas bear a disproportionate burden of traffic-related injuries and air pollution (Sustainable Development Commission, 2011).

The use of active or motorised modes of transport is likely to be influenced by the built environment. Providing new or improved major roads has been shown to increase traffic (Standing Advisory Committee on Trunk Road Assessment, 1994) and, coupled with current patterns of dispersed land use and urban sprawl, contributes to making cars an attractive option (British Medical Association, 2012). By contrast, preliminary evidence suggests that changes to the environment such as traffic calming, road user charging and constructing walking or cycling infrastructure may promote active travel (National Institute for Health and Clinical Excellence (NICE), 2008; Goodman et al., 2014; Panter et al., 2016). This limited body of evidence suggests that infrastructure designed to facilitate motorised transport tends to do so, and correspondingly that infrastructure designed to promote active travel also tends to do so. However, the effects on active travel of major new road infrastructure, for example a new motorway (freeway), are unknown. A systematic review has indicated that new urban roads could contribute to community severance (separation of residents from local amenities or social networks), which might adversely affect active travel; but there was, and remains, no clear evidence from intervention studies in this area (Egan et al., 2003).

The M74 motorway extension in Glasgow, Scotland entailed the construction of an eight kilometre (five mile) section of motorway comprising six lanes through a predominantly urban, deprived area. The new motorway was intended to relieve congestion on the M8, an existing motorway built in the 1960 s which traverses the city centre. Construction of the motorway involved bridging existing local roads and building four new motorway junctions (access points). Advocates of the project argued that relieving congestion would improve local conditions for active travel, whereas opponents countered that the new motorway would simply reinforce car dependence.

The opening of the M74 motorway extension thereby presented an opportunity to examine the impacts of new major road infrastructure on travel behaviour using a natural experimental study. Recently, there have been calls for more evidence of this nature where randomisation is unfeasible, or in this case, impossible (Craig et al., 2012). Therefore, in this study we aimed to evaluate the effects of living near a new urban motorway on travel, and active travel, behaviour in local communities.

## 2 Materials and methods

### 2.1 Design

We examined the effects of the M74 motorway extension on travel behaviour in a natural experimental study of a longitudinal cohort within two distinct cross-sectional samples recruited at baseline (2005) and follow-up (2013). The study received ethical approval from the University of Glasgow (baseline reference FM01304; follow-up reference 400120077).

Further information on the baseline study hypotheses, methods (Ogilvie et al., 2006) and sample characteristics (Ogilvie et al., 2008) can be found elsewhere.

### 2.2 Study areas

To allow for controlled comparisons, we iteratively delineated three study areas in Glasgow using a Geographic Information System (GIS). The study areas were defined as census output areas (the smallest unit for which census data are available) lying wholly or partly within a 500 m buffer surrounding the proposed new M74 motorway extension (South study area), the existing M8 motorway (East study area) and the suburban railway between Cowlairs and Maryhill (North study area containing no motorway).

**Fig. 1:**
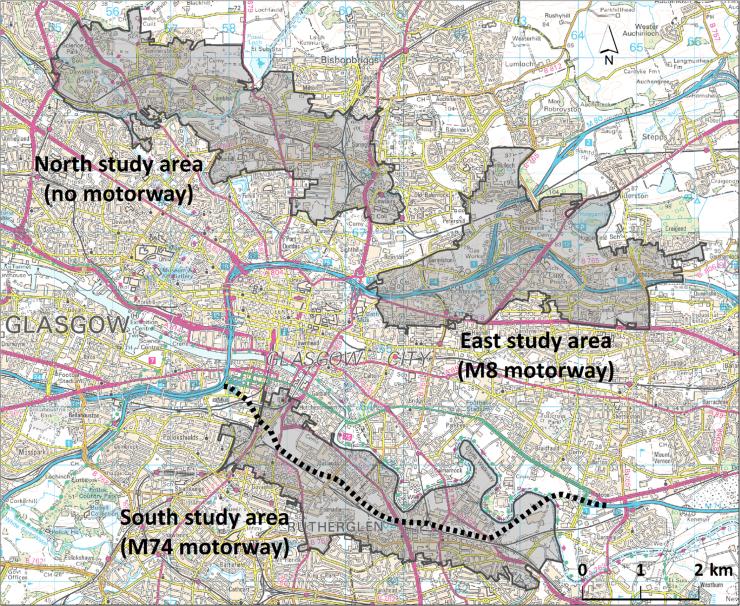
Boundaries of local study areas in Glasgow at baseline (2005) defined in terms of census output areas. Dotted line shows route of M74 motorway extension. Data and raster image © Crown Copyright/database right 2005. An Ordnance Survey/EDINA supplied service.

Aggregate census data, including levels of deprivation and unemployment, home and car ownership, and prevalence of chronic illness, were tabulated for the South (new motorway) study area. The boundaries of the East (existing motorway) and North (no motorway) study areas were then iteratively adjusted using a GIS until they matched the South (new motorway) study area on these characteristics (Table 1). During this process, field visits were conducted to ascertain the general characteristics of the built environment in the three areas, to confirm the face validity of the general comparability of the study areas and aid in producing the final boundaries. All three study areas extended from near the city centre to residential suburbs, contained other major arterial roads, had a mixture of housing stock and had broadly similar socioeconomic characteristics, but differed in their proximity to motorway infrastructure (Fig. 1) (Ogilvie et al., 2008).

### 2.3 Intervention

The M74 motorway extension was built through or close to mainly residential areas and opened in 2011. A pre-construction environmental impact assessment (Scottish Executive, 2003) proposed that the new motorway would relieve congestion on existing motorways and main roads by reducing journey times. Although traffic flows were forecast to increase on feeder roads to the new motorway junctions, the increased efficiency of the motorway network was predicted to divert traffic away from the local road network overall, leading to quieter local roads and improved conditions for active travel and public transport.

However, an independent public local inquiry (Hickman and Watt, 2004) concluded that any benefits on journey times would not be sustained in the long term, and the new motorway would simply encourage the use of, and dependence on, motor vehicles. An overall increase in traffic, as well as the physical presence of the motorway, would degrade the environment, contribute to community severance, and discourage active travel. The inquiry further noted that due to low levels of car ownership, the motorway would be of little use to many local residents and that increasing provision for car users would leave those without cars ultimately more disadvantaged.

In summary, advocates of the motorway argued that it would promote active travel and public transport, whereas opponents argued that it would discourage active travel and promote car use. The authors were independent of all parties making claims about the projected effects of the new motorway.

### 2.4 Sampling and recruitment of participants

Participants were adults aged 16 years or over who responded to a postal survey delivered to their home address. Participants were recruited prior to motorway construction in 2005 (T1), and approximately two years after the motorway opened in 2013 (T2). At each time point, a postal survey was mailed to a random sample of private residential addresses drawn from each of the three study areas using the Royal Mail Postcode Address File. At baseline, participants were given the option to be contacted again in the future. Yearly contact was maintained with those who agreed, and all who could still be contacted were mailed a survey at follow-up. 9,000 surveys in total were mailed at each time point, 3,000 in each study area. In this way we generated a longitudinal cohort and two distinct cross-sectional samples.

A notification postcard preceded each survey, in line with recommendations to maximise response (Edwards et al., 2009). Non-responders were sent the survey again approximately one month after the first mailing. The mailings were seasonally matched, with the initial survey at each time point mailed in October, and staggered over multiple days to encourage responses reflecting different days of the week. Respondents were entered into a £50 ($70) prize draw (baseline) or received a £5 ($7) voucher (follow-up). Responses received more than three months after the first mailing were disregarded.

### 2.5 Measurement

The survey included items on demographic and socioeconomic characteristics, travel behaviour (including a previous-day travel record, see below), physical activity, health and well-being and perceptions of the local neighbourhood.

#### 2.5.1 Travel behaviour

We used a record of all travel made on the previous day which was adapted from similar instruments used in the Scottish Household Survey and the National Travel Survey. For each journey, participants were asked to report the purpose, the mode(s) of transport used and the number of minutes spent using each mode. Both single- and multi-modal journeys could be reported. Participants were asked not to report journeys made in the course of employment (e.g. as a taxi driver, or from an office to attend an external meeting) or journeys made purely for exercise or recreation (e.g. walking the dog).

For analysis, we excluded the travel records of participants who returned a completely blank record, returned a record that was deemed implausible, reported not being at home on the day in question, or returned non-numeric responses such as a tick instead of a number of minutes. We retained records in which participants had reported no journeys but had completed other parts of the record such as specifying the day of the week and whether they had been at home, treating these as “true zero” records indicating no travel. Several participants reported journeys for which the purpose was either not stated or deemed ineligible (employment-related or recreational). All such journeys were deleted before further analysis. Time spent using each mode of transport was summed and used to calculate total time spent travelling and time spent using the bus, using the car and walking, all in minutes per day. Because of the very small proportions of participants using the train or bicycle (less than 6%), summary variables were not derived for these modes.

#### 2.5.2 Exposure

Area-level exposure was defined as residence in the South (new motorway), East (existing motorway) or North (no motorway) study area. In addition, we defined another set of individual-level exposures. Using a GIS, we calculated the distance in metres from the weighted population centroid of the unit postcode for each participant's home address by road network to the nearest motorway junction. Unit postcodes are the smallest unit of postal geography in the UK and contain approximately 15 residential addresses on average. As we hypothesised that the effect of a given change in distance from a motorway would be greater among those who lived very close, we transformed this exposure using the negative natural log. The final measure therefore represented proximity to the motorway, whereby a higher value reflected greater exposure and a unit change in exposure corresponded, for example, to the difference between those living 100 m and 300 m from a motorway, or between those living 300 and 800 m away.

### 2.6 Analysis

Descriptive analyses of the longitudinal cohort and repeat cross-sectional sample were undertaken at baseline (T1) and follow-up (T2). Differences in demographic and socioeconomic characteristics between study areas and across time points were investigated using one-way ANOVA, t and chi-squared tests as appropriate, and those between the longitudinal cohort and the rest of the baseline sample were explored using t and chi-squared tests.

We then undertook two main sets of analysis. The first examined within-participant change over time in the longitudinal cohort (thereby including all participants who provided data at both time points). The second examined population-level change over time in the repeat cross-sectional sample (in which all participants provided data at one time point only, which prohibited the examination of change within individuals).

Preliminary exploration indicated that the assumptions of linear regression could not be satisfied because of non-linearity and skewness; therefore, we used two-part models (Cragg, 1971) to examine the relationship between motorway exposure and travel behaviour. These entail modelling the relationship in two stages: first the likelihood of reporting the behaviour (for example, using the car – yes or no, expressed as a binary outcome), and second the quantity of the behaviour, but only among those who reported it (for example, time spent using the car among those who used the car, expressed as a continuous outcome). For the first stage we used a logit regression. For the second stage we used a generalised linear model (GLM) with a gamma family and log link, because the distribution of the outcome variable remained skewed even after removing the zero values. The resulting incidence rate ratio can be interpreted as the proportional change in the outcome following a one unit increase in the exposure. When using two-part models is it important that zeroes are genuine; in other words, they should reflect people truly not engaging in the behaviour rather than simply not responding to the question. Removing participants who returned a blank travel record during the cleaning procedures described above was intended to satisfy this criterion.

Analyses were carried out using Stata 13 (Timberlake, London, UK) to assess the relationships of (a) study area, and (b) individual-level exposure stratified by study area, with (i) travel and travel time, (ii) bus use and bus time, (iii) car use and car time and (iv) walking and walking time. The final models were adjusted for age, sex, home ownership, car ownership, working status and years lived in the local area. For all analyses using study area as the exposure, we used the North (no motorway) study area as the reference category. In the longitudinal cohort analyses we modelled the outcome as the follow-up value adjusting for the baseline value of that variable, which we interpreted as within-participant change over time. In the cohort analyses using individual-level exposure stratified by study area, we only carried out the first stage of the two-part model (logit regression) due to the small number of non-zero values available for the second stage of the model. For the repeat cross-sectional analyses we added a variable indicating time point, whereby the coefficient of the interaction between the time point and exposure gave an indication of the population shift in the outcome over time.

In the cohort analysis, we tested for interactions by car ownership. Finally, as a sensitivity analysis, we re-ran the repeat cross-sectional analyses using all participants who had provided data at either time point (i.e. both longitudinal and repeat cross-sectional participants).

## 3 Results

### 3.1 Response

1,345 completed surveys were returned at T1 and 1,343 at T2. After accounting for survey packs which could not be delivered (676 at baseline and 509 at follow-up), the response was similar at both time points: 16.1% at T1 and 15.8% at T2. The longitudinal cohort comprised the 365 participants who returned surveys at both time points. The remaining 980 (T1) and 978 (T2) participants together formed the repeat cross-sectional sample. After cleaning, 1,141 travel records in total were suitable for analysis at T1, and 1,206 were suitable at T2. A total of 71 ineligible journeys were removed at T1, and 92 were removed at T2.

### 3.2 Differences between study areas, time points and samples

Table 2 presents descriptive characteristics of the longitudinal and repeat cross-sectional samples. Table 3 presents sociodemographic characteristics and unadjusted summary measures of travel by study area. There were no significant sociodemographic differences between study areas in the longitudinal cohort. However, in the T2 repeat cross-sectional sample, on average participants in the North (no motorway) study area were older, and participants in the South (new motorway) had lived fewer years in their locality, than those in the other areas (there were no significant differences at T1).

In the cohort, significant changes over time in age, working status and duration of residence in the local area were consistent with an ageing sample. In the repeat cross-sectional sample, the members of the T2 sample were on average older, with a higher proportion of men and car owners, than those of the T1 sample. In both the longitudinal and repeat cross-sectional samples, the proportion of participants undertaking any travel, and using any of the various modes of transport analysed, was on average markedly and significantly lower at T2 than at T1 (Table 2).

Compared to the rest of the T1 sample, cohort participants were significantly more likely to be men (43% versus 37%, p=0.034), home owners (61% versus 48%, p<0.001), car owners (58% versus 49%, p=0.002), and to be employed or studying (59% versus 48%, p=0.001). They were correspondingly significantly more likely to travel (90% versus 85%, p=0.033) and to use a car (52% versus 44%, p=0.025) at baseline.

### 3.3 Cohort analysis

The results of the multivariable two-part regression models are displayed in Table 4. Compared to those in the North (no motorway) study area, cohort participants in the South (new motorway) were significantly more likely to undertake travel by any mode at follow-up (odds ratio [OR] 2.1, 95% confidence interval [CI] 1.0–4.2), and those in the East (existing motorway) were significantly more likely to use the bus at follow-up (OR 2.4, 95% CI 1.1–5.2). However, there were no differences between study areas for either time spent travelling in general, or time spent using any mode of transport in particular.

Within the South (new motorway) study area, participants living closer to a motorway junction were more likely to use a car and to undertake travel by any mode at follow-up than those living further away, but only the finding for any travel remained statistically significant in the maximally adjusted model (OR 4.7, 95% CI 1.1–19.7).

Within the East (existing motorway) study area, a significant interaction was found by car ownership. Stratified analysis indicated that in participants who owned a car, those living closer to a motorway junction were more likely to use the bus at follow-up than those living further away (OR 4.5, 95% CI 0.9–21.5), an effect not found in those without a car.

### 3.4 Repeat cross-sectional analysis

The results of the multivariable two-part regression models are displayed in Table 5. There were no significant differences between study areas for either likelihood of, or time spent using, any or all modes of travel. However within the South (new motorway) study area, participants living closer to a motorway junction were more likely to use a car at follow-up than those living further away (OR 3.4, 95% CI 1.1–10.7). The sensitivity analysis did not substantially change these findings.

## 4 Discussion

### 4.1 Main findings

Against a backdrop of a decrease in travel over time, we found some evidence that the new M74 motorway promoted relative increases in travel generally, and car use more specifically, in those living nearby. The findings in cohort participants – an increased likelihood of travel in those living in the general area, and in those living nearer to a motorway junction – suggest that the effects of a new motorway may have been particularly pronounced in a group who were already wealthier and more mobile than the general local population. The new motorway also appeared to promote car use in the local population living near a motorway junction, although not in the cohort analysis. This may reflect the higher prevalence of car ownership at follow-up in the repeat cross-sectional sample, with motorway construction encouraging those with cars to move into the area, or encouraging those already in the area to acquire them. This could be expected to disadvantage the half of our sample who did not own a car. Notably, we did not find any effects (either an increase or a decrease) on active travel, contrary to claims made by both advocates and opponents of the new motorway.

Four-week (Transport Scotland, 2012a), 16-week (Transport Scotland, 2012b) and one-year (Transport Scotland, 2015) evaluations of the new motorway conducted by Transport Scotland indicated marked reductions in traffic flows and journey times across parts of the motorway network, suggesting that the primary objective of relieving congestion was achieved. Decreases in traffic flow on the local road network were also observed, with the exception of local streets leading to the new motorway junctions, where traffic increased. The findings of our study suggest that a major piece of transport infrastructure designed to improve the efficiency of travel and facilitate car use did indeed promote travel and car use. These findings mirror, and are conceptually consistent with, previous work which suggests that creating new active travel infrastructure can promote active travel (National Institute for Health and Clinical Excellence (NICE), 2008; Goodman et al., 2014; Panter et al., 2016).

### 4.2 Strengths and limitations

This study adds to a small but growing body of evidence examining how changes in the built environment influence changes in travel behaviour, in accordance with calls for more evidence of this nature (National Institute for Health and Clinical Excellence (NICE), 2008; Craig et al., 2012). This study is one of very few examining the effects of environmental interventions on active living in deprived populations. We used a travel record that allowed us to disaggregate individual travel modes, and objectively defined area and individual-level exposures using a GIS. We controlled for a series of potential confounders in all models in a combination of longitudinal and repeat cross-sectional analyses, and used two-part models, which have seldom been used in this field but are an efficient way of combining analytical options.

The limitations of our study include the collection of only one day of travel data, which raises the possibility that travel on a given sampled day was not typical and increases the variability in the data. There was a comparatively low response to the survey, which — coupled with missing data on the travel diary — limits the external validity of the findings, although our response rate was not unusual for this type of natural experimental study (Sahlqvist et al., 2011; Cummins et al., 2005). We chose to include a repeat cross-sectional design because we anticipated considerable attrition of the cohort, and this was confirmed, with approximately 70% of the original baseline sample lost after eight years of follow-up. However, again this was comparable to that of other similar studies (Goodman et al., 2014; Egan et al., 2013). The marked reductions in travel evident at follow-up may have been an artefact of measurement, or a reflection of a real decline, or both. Importantly, this decline was consistent across samples and study areas, which suggests our examination of intervention effects using relative comparisons by study area and proximity is still valid. The cohort and repeat cross-sectional analyses did not fully correspond. However, the approaches differ in terms of examining individual and population-level effects respectively, and it is likely the intervention operated differently at these levels. Finally, the possibility of unmeasured confounding related to other regeneration projects or concurrent changes in the built environment is a core challenge of this type of natural experimental research, and is further elaborated below.

### 4.3 Implications for research

The finding of an increased likelihood of bus use over time in cohort participants living in the study area containing the existing M8 motorway, and in car owners living near an M8 motorway junction, highlights the issue of competing interventions and unmeasured confounding in natural experimental research. Causal attribution of increasing bus use between 2005 and 2013 to a motorway built in the 1960 s seemed unlikely. Rather, it seems more likely that these findings reflected concurrent improvements to bus network provision in the East (existing motorway) study area. In particular, car owners may have a greater choice of transport mode than non-car owners, and thus may be more amenable to changing modes when public transport provision improves. In contrast, non-car owners may be more constrained in their travel choices and need to use public transport regardless of improvements in provision. This interpretation is further supported by the fact that bus services do run on the M8 motorway (and on other motorways in Glasgow, including the M74), though these are not generally serving local journeys.

An initial scoping exercise indicated that public transport improvements had taken place in this area, but the scale of work required to comprehensively assess and quantify this additional time-varying exposure was outwith of the scope of the current study. Therefore, while assessing the ‘face validity’ of causal statements and identifying potential competing interventions is an important step toward enhancing the internal validity of natural experimental studies, delineating these competing interventions can be as complex as delineating the intervention under study, if not more so.

### 4.4 Implications for policy

Car ownership is patterned by deprivation, with lower ownership in lower income groups (Dargay, 2001). In this study, approximately 50% of our repeat cross-sectional sample and 40% of our cohort did not own a car, compared with a Scottish national average of 29% in 2011/12 (Transport Scotland, 2014). In Scotland, lower income is associated with less travel by car, a lower number of journeys and lower overall distance travelled, and not owning a car is also associated with a lower number of journeys (Transport Scotland, 2014).

The new M74 motorway appeared to promote travel and car use, and thus may have contributed to existing socioeconomic inequalities relating to income and car ownership in local communities. This is despite a stated commitment by the Scottish Government to invest in sustainable modes of transport and to increase the accessibility of the transport network to disadvantaged communities in particular (Transport Scotland, 2016), which is echoed further in regional transport policy documents (Strathclyde Partnership for Transport, 2014). The results of this study suggest a potentially inequitable distribution of the benefits of the new motorway on traffic congestion and journey times in local communities. Emerging findings from a complementary body of qualitative research suggests that car owners differed from non-car owners in terms of their experience of the new motorway as a connecting or severing force, and data from a second complementary investigation, in which a subsample of study participants underwent objective activity monitoring using accelerometers and global positioning system receivers, is currently being analysed. These findings will be explored in detail in future reports before compiling a more comprehensive picture to help guide future policy on investing in major transport infrastructure.

### 4.5 Conclusions

The new M74 motorway appeared to promote travel and car use in those living nearby, but did not influence active travel behaviour. It therefore has the potential to reinforce existing socioeconomic inequalities in health, particularly for those who do not own a car.

## Competing interests

LF reports grants from National Institute for Health Research Public Health Research programme, project number 11/3005/07, grants from British Heart Foundation, Cancer Research UK, Economic and Social Research Council, Medical Research Council, the National Institute for Health Research, and the Wellcome Trust, under the auspices of the UK Clinical Research Collaboration (UKCRC), during the conduct of the study; RP reports grants from Medical Research Council, grants from National Institute for Health Research, during the conduct of the study; FC has nothing to disclose; SS has nothing to disclose; DO reports grants from Medical Research Council, grants from NIHR Public Health Research Programme, grants from UKCRC, during the conduct of the study.

## Figures and Tables

**Table 1:** Aggregate sociodemographic characteristics of study areas using 2001 census data.

**Variable**	**South**	**East**	**North**
Households (number)	10059	12227	11808
% home ownership^a^	38	39	38
% no access to a car^a^	66	66	64
% with chronic illness^b^	28	27	27
% male unemployment^c^	9	10	9
% top three NS-SEC categories^d^	15	12	14
% usually travel to work by car^e^	45	44	45

NS-SEC – National Statistics Socio-economic Classification.

North – study area containing no motorway infrastructure; East – study area containing existing M8 motorway; South – study area containing new M74 motorway.

a Denominator: households.

b Denominator: ‘working age’ population (men aged 16–64 and women aged 16–59).

c Denominator: men aged 16–74.

d Denominator: population aged 16–74.

e Denominator: population who travel to place of work or study.

**Table 2:** Characteristics of the longitudinal cohort and repeat cross-sectional sample. Data collected in Glasgow at T1 (2005) and T2 (2013).

**Variable**	**Longitudinal cohort** (n=365)				**Repeat cross-sectional sample** (T1 n=980; T2 n=978)			
	T1		T2		T1		T2	
	*n*	*mean (SD) /%*	*n*	*mean (SD) /%*	*n*	*mean (SD) /%*	*n*	*mean (SD) /%*
Age (years)	360	50.4 (13.6)	363	58.5 (13.6)**	962	48.8 (18.3)	970	52.6 (16.5)**
% male	361	43.5	363	44.4	970	37.1	972	42.8**
% home ownership	360	61.1	363	62.5	965	47.9	971	49.6
% car ownership	361	58.5	362	60.5	951	48.8	969	53.4**
% working*	359	58.5	364	48.1**	961	48.3	972	48.3
Years lived in local area	365	18.3 (15.3)	362	24.9 (16.6)**	980	18.2 (18.0)	965	19.0 (17.4)
% travelled	285	90.5	285	68.8**	830	84.8	877	65.0**
Travel time if travelled (min/day)	258	76.1 (52.3)	196	75.1 (81.5)†	704	67.1 (50.9)	570	67.4 (57.8)
% used the bus	285	31.9	285	21.1**	830	31.7	877	23.3**
Bus time if used the bus (min/day)	91	52.4 (44.9)	60	47.4 (35.0)†	263	42.2 (36.8)	204	49.5 (53.4)
% used the car	285	52.6	285	41.8**	830	44.3	877	34.5**
Car time if used the car (min/day)	150	53.0 (43.0)	119	50.1 (46.4)†	368	50.2 (47.4)	303	49.3 (44.3)
% walked	285	56.5	285	36.1**	830	53.1	877	33.1**
Walk time if walked (min/day)	161	35.3 (27.1)	103	37.7 (27.7)†	441	35.3 (30.2)	290	34.7 (30.1)

min – minutes; n – number; T – time point; SD – standard deviation.

* In paid employment (full or part-time), full-time student, or undertaking voluntary work.

** Significant difference between time points within the same study sample (p<0.05).

† Did not test for differences between time points because of small sample.

**Table 3:** Sociodemographic characteristics and unadjusted measures of travel behaviour by study area and time point. Data collected in Glasgow at T1 (2005) and T2 (2013).

**Variable**	**Longitudinal cohort** (n=365)				**Repeat cross-sectional sample** (T1 n=980; T2 n=978)			
	T1		T2		T1		T2	
	*n*	*mean (SD) /%*	*n*	*mean (SD) /%*	*n*	*mean (SD) /%*	*n*	*mean (SD) /%*
Age (years)								
Total	360	50.4 (13.6)	363	58.5 (13.6)	962	48.8 (18.3)	970	52.6 (16.5)
North	124	49.0 (13.3)	126	57.3 (13.4)	333	49.7 (18.2)	337	54.6 (16.0)
East	111	51.3 (13.3)	112	59.4 (13.3)	317	48.5 (18.7)	329	51.8 (17.0)
South	125	51.0 (14.1)	125	59.0 (14.1)	312	48.1 (17.8)	304	51.2 (16.4)

% male								
Total	361	43.5	363	44.4	970	37.1	972	42.8
North	125	37.6	126	38.9	337	36.2	337	43.3
East	111	44.1	112	44.6	318	34.0	331	40.2
South	125	48.8	125	49.6	315	41.3	304	45.1

% home ownership								
Total	360	61.1	363	62.5	965	47.9	971	49.6
North	125	60.8	126	62.7	337	46.3	336	50.3
East	111	61.3	112	62.5	313	51.1	331	48.6
South	124	61.3	125	62.4	315	46.4	304	50.0

% car ownership								
Total	361	58.5	362	60.5	951	48.8	969	53.4
North	125	61.6	126	65.9	332	49.4	336	54.8
East	111	52.3	112	55.4	312	49.4	329	52.3
South	125	60.8	124	59.7	307	47.6	304	53.0

% working^a^								
Total	359	58.5	364	48.1	961	48.3	972	48.3
North	125	60.8	127	50.4	333	47.2	338	44.4
East	110	54.6	112	46.4	315	48.9	330	49.7
South	124	59.7	125	47.2	313	48.9	304	51.0

Years lived in local area								
Total	365	18.3 (15.3)	362	24.9 (16.6)	980	18.2 (18.0)	965	19.0 (17.4)
North	127	16.9 (13.1)	126	22.7 (14.1)	338	18.9 (18.7)	332	19.7 (16.9)
East	112	17.5 (13.5)	110	24.9 (14.0)	319	18.2 (16.9)	330	20.7 (18.1)
South	126	20.3 (18.4)	126	27.0 (20.3)	323	17.3 (18.4)	303	16.3 (17.1)

% travelled								
Total	285	90.5	285	68.8	830	84.8	877	65.0
North	101	88.1	101	64.4	285	84.6	306	61.4
East	87	94.3	87	70.1	267	86.9	300	67.0
South	97	89.7	97	72.2	278	83.1	271	66.8

Travel time if travelled (min/day)								
Total	258	76.1 (52.3)	196	75.1 (81.5)	704	67.1 (50.9)	570	67.4 (57.8)
North	89	75.3 (52.7)	65	83.9 (118.8)	241	66.7 (50.3)	188	67.1 (53.1)
East	82	76.5 (47.4)	61	80.3 (65.3)	232	62.7 (40.4)	201	68.9 (58.8)
South	87	76.6 (56.7)	70	62.2 (41.2)	231	71.9 (59.9)	181	66.1 (61.5)

% used the bus								
Total	285	31.9	285	21.1	830	31.7	877	23.3
North	101	27.7	101	14.9	285	31.2	306	20.9
East	87	41.4	87	32.2	267	34.1	300	27.3
South	97	27.8	97	17.5	278	29.9	271	21.4

Bus travel time if used the bus (min/day)								
Total	91	52.4 (44.9)	60	47.4 (35.0)	263	42.2 (36.8)	204	49.5 (53.4)
North	28	46.1 (43.0)	15	47.2 (30.1)	89	44.3 (38.5)	64	48.0 (60.7)
East	36	62.0 (48.1)	28	47.6 (38.3)	91	36.8 (28.2)	82	53.7 (54.0)
South	27	46.0 (41.4)	17	47.1 (35.3)	83	45.8 (42.5)	58	45.4 (43.5)

% used the car								
Total	285	52.6	285	41.8	830	44.3	877	34.6
North	101	57.4	101	42.6	285	45.3	306	34.3
East	87	43.7	87	36.8	267	46.1	300	33.3
South	97	55.7	97	45.4	278	41.7	271	36.2

Car travel time if used the car (min/day)								
Total	150	53.0 (43.0)	119	50.1 (46.4)	368	50.2 (47.4)	303	49.3 (44.3)
North	58	52.7 (47.5)	43	51.4 (44.8)	129	48.0 (40.3)	105	51.5 (39.9)
East	38	54.9 (40.5)	32	53.3 (59.3)	123	47.0 (35.4)	100	48.6 (41.9)
South	54	52.1(40.4)	44	46.6 (37.1)	116	56.0 (63.2)	98	47.7 (50.9)

% walked								
Total	285	56.5	285	36.1	830	53.1	877	33.1
North	101	53.5	101	32.7	285	51.6	306	31.4
East	87	58.6	87	41.4	267	51.7	300	32.7
South	97	57.7	97	35.1	278	56.1	271	35.4

Walking travel time if walked (min/day)								
Total	161	35.3 (27.1)	103	37.7 (27.7)	441	35.3 (30.2)	290	34.7 (30.1)
North	54	35.8 (28.5)	33	35.6 (25.1)	147	33.5 (31.4)	96	33.6 (28.4)
East	51	32.9 (25.8)	36	41.5 (28.9)	138	35.4 (27.5)	98	35.2 (27.6)
South	56	37.0 (27.1)	34	35.9 (29.0)	156	36.8 (31.4)	96	35.1 (34.1)

min – minutes; n – number; T – time point; SD – standard deviation.

North – study area containing no motorway infrastructure; East – study area containing existing M8 motorway; South – study area containing new M74 motorway.

a In paid employment (full or part-time), full-time student, or undertaking voluntary work.

**Table 4:** Longitudinal associations between exposure to a motorway and change in travel behaviour. Data collected in Glasgow at T1 (2005) and T2 (2013).

**Exposure**	**Travel**				**Bus**				**Car**				**Walking**			
	*n*	*yes/no*	*n*	*min /day*	*n*	*yes/no*	*n*	*min /day*	*n*	*yes/no*	*n*	*min /day*	*n*	*yes/no*	*n*	*min /day*
		*OR (95% CI)*		*IRR (95%CI)*		*OR (95%CI)*		*IRR (95%CI)*		*OR (95%CI)*		*IRR (95%CI)*		*OR (95%CI)*		*IRR (95%CI)*
**Area**: East (reference: North)	277	1.8 (0.9, 3.6)	193	1.0 (0.7, 1.5)	277	2.4 (1.1, 5.2)*	59	1.1 (0.7, 1.7)	277	1.1 (0.6, 2.2)	119	1.0 (0.7, 1.6)	277	1.6 (0.8, 3.1)	100	1.4 (1.0, 2.0)
**Proximity** within East study area	83	1.6 (0.6, 3.9)	–	–	83	1.3 (0.6, 3.0)	–	–	83	1.2 (0.5, 3.0)	–	–	83	1.7 (0.8, 3.6)	–	–
**Area**: South (reference: North)	277	2.1 (1.0, 4.2)*	193	0.8 (0.5, 1.1)	277	1.3 (0.6, 3.0)	59	1.0 (0.6, 1.7)	277	1.4 (0.7, 2.7)	119	0.9 (0.6, 1.3)	277	1.2 (0.6, 2.3)	100	0.9 (0.6, 1.4)
**Proximity** within South study area	91	4.7 (1.1, 19.7)*	–	–	91	2.1 (0.3, 13.1)	–	–	91	2.3 (0.7, 8.1)	–	–	91	2.0 (0.5, 7.6)	–	–

CI – confidence interval; IRR – incidence rate ratio; min – minutes; n – number; OR – odds ratio.

Two-part model adjusted for age, sex, home ownership, car ownership, working status, years lived in the local area and baseline value of the outcome of the model in question.

Sample comprised study participants who provided data at both time points.

North – study area containing no motorway infrastructure; East – study area containing existing M8 motorway; South – study area containing new M74 motorway.

* p<0.05.

**Table 5:** Repeat cross-sectional associations between exposure to a motorway and change in travel behaviour. Data collected in Glasgow at T1 (2005) and T2 (2013).

**Exposure**	**Travel**				**Bus**				**Car**				**Walking**			
	*obs*	*yes/no*	*obs*	*min /day*	*obs*	*yes/no*	*obs*	*min /day*	*obs*	*yes/no*	*obs*	*min /day*	*obs*	*yes/no*	*obs*	*min /day*
		*OR (95%CI)*		*IRR (95%CI)*		*OR (95%CI)*		*IRR (95%CI)*		*OR (95%CI)*		*IRR (95%CI)*		*OR (95%CI)*		*IRR (95%CI)*
**Area**: East (reference: North)	1655	0.9 (0.4, 1.6)	1252	1.1 (0.9, 1.3)	1655	1.2 (0.7, 2.1)	451	1.3 (0.9, 2.0)	1655	0.8 (0.4, 1.5)	669	1.0 (0.7, 1.3)	1655	0.9 (0.6, 1.5)	717	1.0 (0.7, 1.4)
**Proximity** within East study area	548	0.7 (0.3, 1.8)	424	1.1 (0.5, 1.5)	548	0.8 (0.4, 1.7)	165	0.8 (0.5, 1.2)	548	1.0 (0.4, 2.3)	223	1.4 (0.9, 2.2)	548	0.7 (0.3, 1.5)	230	1.4 (0.8, 2.3)
**Area**: South (reference: North)	1655	1.0 (0.5, 1.9)	1252	0.9 (0.7, 1.1)	1655	1.0 (0.6, 1.8)	451	0.9 (0.6, 1.3)	1655	1.1 (0.6, 2.0)	669	0.7 (0.5, 1.0)	1655	0.8 (0.5, 1.4)	717	0.9 (0.7, 1.3)
**Proximity** within South study area	534	0.8 (0.3, 2.7)	406	1.3 (0.9, 2.1)	534	0.9 (0.3, 2.4)	140	1.9 (0.8, 4.3)	534	3.4 (1.1, 10.7)*	212	1.1 (0.5, 2.3)	534	1.1 (0.5, 2.7)	249	1.2 (0.7, 2.0)

CI – confidence interval; IRR – incidence rate ratio; min – minutes; obs – observations; OR – odds ratio.

Two-part model adjusted for age, sex, home ownership, car ownership, working status and years lived in the local area.

Sample comprised study participants who provided data at one time point only.

North – study area containing no motorway infrastructure; East – study area containing existing M8 motorway; South – study area containing new M74 motorway.

* p<0.05.
